# Efficacy and safety of a single radiofrequency ablation of solid benign non-functioning thyroid nodules

**DOI:** 10.1590/2359-3997000000246

**Published:** 2017-01-27

**Authors:** Roberto Cesareo, Andrea Palermo, Valerio Pasqualini, Carla Simeoni, Alessandro Casini, Giuseppe Pelle, Silvia Manfrini, Giuseppe Campagna, Roberto Cianni

**Affiliations:** 1 Department of Internal Medicine “S. M. Goretti” Hospital Latina Italy Department of Internal Medicine “S. M. Goretti” Hospital, Latina, Italy; 2 Department of Endocrinology University Campus Bio-Medico Rome Italy Department of Endocrinology, University Campus Bio-Medico, Rome, Italy; 3 Department of Radiology “S. M. Goretti” Hospital Latina Italy Department of Radiology, “S. M. Goretti” Hospital, Latina, Italy; 4 Compensatory authority Rome Italy Compensatory authority (INAIL), Monte Porzio Catone, Rome, Italy

**Keywords:** Radiofrequency ablation, thyroid nodules, ultrasounds, cosmetic score, symptom score

## Abstract

**Objective:**

The objective of our study is to evaluate the clinical outcomes and safety of radiofrequency thermal ablation (RFA) for benign thyroid nodules (BTNs) over a 1-year follow-up.

**Subjects and methods:**

This is a monocentric retrospective study. Forty-eight patients with solid, non-functioning BTNs were treated by RFA using a 17G internally cooled electrode. We categorized thyroid nodules as small (≤ 12 mL), medium (12 to 30 mL), or large (over 30 mL). BTNs volume reduction, thyroid function, cosmetic and compressive score changes and side effect evaluation at 6 and 12 months were evaluated.

**Results:**

BTN volume decreased significantly from baseline to 6 (mean percentage decrease of BTN volume was 66.8 ± 13.6%, p < 0.001). At 12 months, the mean percentage reduction of BTN volume compared to six months was 13.7 ± 17.1% (p < 0.001). At 6-month, symptom score had improved significantly (p < 0.001) while it does not change significantly between 6 and 12 months. In particular, symptom score improved significantly in the medium (p < 0.001) and large (p < 0.01) subgroups. Cosmetic score improved significantly between baseline and 6 months (p < 0.001) and between 6 and 12 months (p < 0.01). In all the subgroups, cosmetic score improved significantly between baseline and 6 months, while between 6 and 12 months it improved significantly only in the large group (p < 0.05). RFA was well tolerated. Only one patient experienced permanent right paramedian vocal cord palsy.

**Conclusions:**

A single RFA treatment was effective in reducing BTNs volume, in particular small and medium nodules. Cosmetic score improved in all treated BTNs while symptom score only got better in the medium and large BTNs.

## INTRODUCTION

Over the last decade, non-surgical minimally invasive US-guided debulking techniques have been proposed to reduce the volume of thyroid nodules when surgery is contraindicated or refused (1). US-guided percutaneous ethanol injection (PEI) therapy is currently considered one of the first-line treatment modality for cystic and predominantly cystic nodules (2,3). Laser ablation (LA) has been proposed as a safe outpatient procedure that effectively reduces the volume of solid nodules and the clinical outcomes in the majority of patients (4-9).

Although it is a newer technique compared to LA, radiofrequency ablation (RFA) has been gaining popularity as a minimally invasive treatment for thyroid nodules (10-12). Indications for RFA of benign thyroid nodules (BTNs) are nodule-related clinical symptoms such as neck pain, dysphasia, foreign body sensation, discomfort, cough, cosmetic problems, or thyrotoxicosis in cases of autonomously functioning thyroid nodules (10-12).

The real impact of RFA of benign thyroid nodules in terms of efficacy is still controversial because most of the published studies are compromised by several bias such as trials not controlled, small thyroid nodule volume or short follow-up period (13-20). Moreover, a few authors have treated large thyroid nodules and, in these patients, further RFA treatments were needed to obtain a significant volumetric reduction (14,15).

Furthermore, there are contrasting evidence on the role of thyroid nodule function (15,16,21), as it may affect the volumetric response to the RFA treatment.

Two retrospective studies have shown the ability of the RFA to reduce the thyroid nodule volume but the authors enrolled patients with cystic nodules and sometimes re-treatment was provided (22,23).

Our retrospective study aims to evaluate the clinical outcomes and safety of a single RFA treatment for benign non-functioning thyroid solid nodules over a 1-year follow-up. In particular, we categorized thyroid nodules in small (≤ 12 mL), medium (12 to 30 mL), or large (over 30 mL) and BTN volume reduction, thyroid function, cosmetic and compressive score changes and side effects were evaluated.

## SUBJECTS AND METHODS

### Study population

From June 2012 to November 2015, a total of 90 patients affected by thyroid nodules were treated and enrolled. Ninety thyroid nodules were treated with US-guided RF ablation at the Thyroid Center of “S. M. Goretti” Hospital.

Subjects were enrolled if they fulfilled all the following criteria:

– Older than 18 years;

– Reported cosmetic and/or symptomatic problems;

– Solid thyroid nodule (solid portion over 70%);

– Thyroid nodules with maximum diameter > 2 cm steadily growing over time;

– Cytologically confirmed benign nodule on two separate US-guided FNAB;

– Serum thyroid hormone (free T4 and free T3), thyrotropin levels (TSH), calcitonin, thyroid peroxidase and thyroglobulin antibodies within normal ranges;

– No history of radioiodine therapy or thermal ablation;

– No previous neck or trunk external beam radiotherapy;

– Refusal of or ineligible for surgery;

– One single RFA treatment.

Exclusion criteria were:

– Pregnancy;

– Malignant or suspicious thyroid nodules;

– Nodules that were confluent in a compressive lobar mass;

– Hot nodule at 99mTc-pertechnetate scintigraphy.

Subjects with small nodules and cosmetic score less than 3 (see section below on the Thyroid nodule classification according to the volume).

Finally, we enrolled 48 patients (17 men, 31 women; age 57.7 ± 14 yrs, range 24-80) who were followed up for 1 year after treatment.

### Thyroid nodule classification according to the volume

We classified nodules according to baseline volume as small (≤ 12 mL), medium (12 to 30 mL), or large (over 30 mL), as previously shown in a recent randomized control trial (24). One single nodule per patient was treated with RFA. In patients with multiple thyroid nodules, the largest and/or most symptomatic one was treated. All patients were clinically, biochemically, and morphologically evaluated at 6 and 12 months.

### Procedure

US was performed using a 7.5–12MHz linear probe equipped with Color Doppler and Power Doppler modules (Technos MPX; Esaote My Lab 50, Italy). We recalculated the nodule volume and percentage of volume reduction (PVR) with the following equations: volume percentage (ellipsoid equation): V = length x width x depth x 0.525; volume reduction percentage: PVR = [initial volume – final volume] ×100)/initial volume (24).

A single BTN volume was measured in case of uninodular goiter or in case of multinodular goiter when characterized by one predominant nodule associated with other non-clinically significant thyroid nodules. We took photos of all enrolled patients at baseline and at 1, 6 and 12 months after RFA. A radiofrequency generator (Cool-tip, E-Series Covidien) and a 17 gauge, 15 cm electrode with a 1 cm active tip was used. All RF procedures were carried out by the same operator under US control with the same scanner used for the initial diagnostic evaluation. The intra- and inter-observer coefficients of variation for sonographic volume assessment were previously defined as 4% and 6%, respectively (24). The patients were treated with 2% Mepivacain 2-5 mL (Carbosen) and 3 mL of Ropivacaine (Naropine, Fresenius Kabi, USA) for local anesthesia at the puncture site. Four mg of dexamethasone IV before RFA of large thyroid nodule were prescribed to reduce post-treatment oedema. On the basis of previous experience, the procedure utilized included the trans-isthmic approach along the short axis of the nodule, and the nodules were managed with the “moving-shot technique” as described elsewhere by Baek and cols. (14).

We adopted a variant of the aforementioned technique, using 60 W of radiofrequency outpower and exposure time needed to obtain a transient multiple hyperechoic zones as a sign of the manoeuvre. All the patients were informed and treated after written informed consent was obtained.

#### Clinical evaluation

We classified symptom and cosmetic scores, as described in a previous consensus statement (10). All patients were asked to rate pressure symptoms on a 10-cm visual analogue scale (grade 0-10 cm) at enrolment and during follow-up. A cosmetic score was obtained according to the following scale: 1, no palpable mass; 2, no cosmetic problem but palpable mass; 3, a cosmetic problem on swallowing only; 4, easily visible mass. According to this classification, we decide to treat subjects with small nodules with cosmetic score more than 2 or nodules with symptoms score > 3.

#### Biochemical evaluation

The laboratory studies included chemiluminescent enzyme immunoassay (Architect i4000 SR, Abbott) for serum thyrotropin (normal range, 0.5–4.9 mIU/L), serum free triiodothyronine (normal range, 1.7-3.7 pg/mL), serum-free thyroxine (normal range, 0.7-1.7 pg/mL), and serum antithyroid peroxidase antibodies (normal range, 0–35 IU/mL); immunoradiometric assay (Architect i4000 SR, Abbott) for serum calcitonin (normal range, 0–10 pg/mL) and blood coagulation tests (prothrombin time, activated partial thromboplastin time).

## Statistical analysis

Statistical analysis was performed using IBM-SPSS Statistics version 21. Descriptive statistics (median, mean, standard deviation, range) were computed on thyroid volume and other clinical variables. To compare group mean values, appropriate parametric test of statistical significance (*t* test) was used, where a Shapiro–Wilk test indicated that the data conformed to a log-normal distribution. Otherwise, an equivalent non parametric test was employed (Kruskal–Wallis test, Wilcoxon for paired sample). The significance level was defined as *p* ≤ 0.05.

## RESULTS

### Nodule volume

Characteristics and clinical data are summarized in Table 1. BTN volume decreased significantly (Table 2) from baseline to 6 months (23.5 ± 18.6 at baseline to 8.5 ± 9 at 6 months; p < 0.001); the mean percentage decrease of BTN volume was 66.8 ± 13.6% at 6 months.

**Table 1:** Main characteristics of the study population and clinical data at baseline

Parameter	

N	48
Sex (males/females)	17/27
Age in years	56 ± 14 (24 – 80)
Thyroid nodule volume (mL)	23.5 ± 18.6 (3.4 – 89)
TSH (mIU/mL)	2.0 ± 0.9 (0.6 – 4.1)
FT3 (pg/mL)	2.6 ± 0.6 (1.2 – 3.7)
FT4 (pg/mL)	1.3 ± 0.2 (0.8 – 1.7)

**Table 2:** Thyroid nodule volume (mL) in radiofrequency ablation group

	Baseline		6 months		12 months
Whole group (n = 48)					
TN vol.	23.5 ± 18.6		8.5 ± 9.0***		7.6 ± 8.7***
TN vol. variation (%) from baseline TN vol. variation (%) from 6 months			-66.8 ± 13.6		-71.1 ± 14.3 -13.7 ± 17.1
Small (n = 12)					
TN vol.	7.4 ± 2.6		2.0 ± 1.1***		1.6 ± 0.9*
TN vol. variation (%) from baseline TN vol. variation (%) from 6 months			-73.5 ± 10.8		-78.7 ± 1 -18.7 ± 18.8
Medium (n = 24)					
TN vol.	18.3 ± 42		6.2 ± 2.6***		5.6 ± 2.7***
TN vol. variation (%) from baseline TN vol. variation (%) from 6 months			-65.8 ± 13.7		-69.0 ± 14.4 -10.8 ± 13.4
Large (n = 12)					
TN vol.	49.8 ± 18.4		19.7 ± 11.6***		17.5 ± 12.3*
TN vol. variation (%) from baseline TN vol. variation (%) from 6 months			-62.0 ± 14.3		-67.7 ± 15.9 -14.7 ± 21.8

Differences are considered between baseline and 6 months and between 6 and 12 months.

* p ≤ 0.05; *** p ≤ 0.001.

At 12 months, BTN volume was 7.6 ± 8.7 mL and the mean percentage reduction of BTN volume compared to six months was 13.7 ± 17.1% (p < 0.001).

The mean percentage of volumetric reduction differed in the three classes of nodules: for small nodules, the mean percentage decrease was 73.5 ± 10.8% at 6 months and 78.7 ± 10% at 12 months; for medium nodules, it was 65.8 ± 13.7% at 6 months and 69 ± 14.4% at 12 months and for large nodules it was 62 ± 14.3% at 6 months and 67.7 ± 15.9% at 12 months.

### Hormonal evaluation

All patients were euthyroid at baseline (Table 1) and serum thyroid function tests after 1, 6 and 12 months did not show significant modification. No significant changes were observed either in TgAb and TPOAb titers or in calcitonin serum concentrations during the follow-up period except for two patients who developed autoimmune thyroid diseases with hyperthyroidism six and twelve months after RF ablation.

### Symptom and cosmetic score evaluation

At 6-month evaluation (Table 3), symptom score improved significantly (p < 0.001) while it doesn’t change significantly between 6 and 12 months (0.5 ± 0.8 at 6 months and 0.4 ± 0.8 at 12 months (p = ns). Between baseline and 6 months, symptom score significantly improved in the medium (p < 0.001) and large subgroups (p < 0.01), whereas, of course, no improvement was observed in the small subgroup. The overall cosmetic score improved significantly between baseline and 6 months (p < 0.001) and between 6 and 12 months (p < 0.01). In all subgroups cosmetic score improved significantly between baseline and 6 months, while between 6 and 12 months it decreased significantly only in large group (p < 0.05).

**Table 3:** Cosmetic score and symptom score groups

	All		Small n = 12		Medium n = 24			Large n = 12	

	Cosmetic score	Symptom score	Cosmetic score	Symptom score	Cosmetic score	Symptom score		Cosmetic score	Symptom score
Baseline	2.8 ± 0.7	3.4 ± 3	3 ± 0	0 ± 0	2.5 ± 0.7		3.6 ± 2.8	3.3 ± 0.8	6.2 ± 1.3
6 months	1.6 ± 0.6***	0.5 ± 0.8***	1.0 ± 0**	0 ± 0	1.6 ± 0.6***		0.4 ± 0.9***	2.2 ± 0.6**	1.0 ± 1.0**
12 months	1.5 ± 0.7**	0.4 ± 0.8°	1.0 ± 0	0 ± 0	1.6 ± 0.6		0.3 ± 0.8°	1.8 ± 0.8*	0.9 ± 1.0°

Differences are considered between baseline and 6 months and between 6 and 12 months.

* p ≤ 0.05; ** p ≤ 0.01; *** p ≤ 0.001; °n.s.

### Complications and safety

RFA was generally safe and well tolerated in all patients, who were placed under observation for 4 hours after the procedure. No patient needed hospitalization after treatment. During the RFA procedure, 8 (21%) of the 48 patients experienced mild local pain, occasionally radiating to the ear or jaw or chest, but it was limited and resolved quickly after the power was switched off. In one patient, however, the procedure was stopped due to severe chest pain (it was resolved quickly after the power was switched off). The most feared complication is voice change after RFA. Only 2 patients (4.7%) had voice change immediately after the RFA session but it resolved completely 2 or 3 hours after the procedure. One patient experienced permanent right paramedian vocal cord palsy with inspiratory stridor without dysphonia.

## DISCUSSION

This one year retrospective study has demonstrated that one single RFA treatment was effective in reducing benign non-functional thyroid nodules volume in particular small and medium nodules.

In order to avoid some confounding factors, we previously excluded from our analysis patients with thyroid cystic nodule or thyroid nodules with a solid portion less than 70%. Indeed a large quantity of fluid may affect the “volumetric” response to RFA and PEI should be used to manage thyroid cystic lesions (2,3).

Up to now, there is no univocal and shared classification that divides the thyroid nodules according to size. Compressive symptoms are usually linked to large nodules, while cosmetic problems can be due to smaller nodules that are located in a superficial part of the gland. In this study, as previously reported (24), we arbitrarily divided the thyroid nodules into small, medium and large (see materials and methods section). According to this distribution, we have noticed that the percentage decrease for the small nodules was larger compared to the other groups, as already shown by other authors (15,23,25).

In particular, according to these findings, multi-RFA treatments would be needed to achieve a clinically significant volume reduction for larger nodules (15,26) therefore, when not contraindicated, surgical treatment or more than a single RFA treatment should be recommended.

We have obtained the maximum volume reduction after 6-month treatment even if we have recorded a further reduction (14,16) at the end of the study period. Only 2 subjects have experienced an increase of the thyroid volume from 6 to 12 months (11.6 mL vs 12.6 mL and 17.3 mL vs 20.0 mL) but none of them exceeded 50% at 1-year post RFA treatment (volume increases after the last follow-up of 8.6 and 15.6% respectively). In another retrospective study, the authors found that 5.6 % subjects (7/126) experienced > 50% increase in nodule volume compared to the previous follow-up volume (23). Probably, the longer follow-up period (4 years) of the Hyun’s study (23) can explain the difference in terms of regrowth rate.

Moreover, our study population is homogenous. Indeed, as previously reported (20,24), thyroid nodules have been treated by one single RFA using the same radiofrequency outpower in order to better evaluate the efficacy of this technique.

We enrolled only patients with non-functioning thyroid nodules because it cannot be fully excluded that their functional state could affect the volumetric response to the RFA treatment (15,16,21,27).

Only two other retrospective studies were published investigating the efficacy of RFA on thyroid nodules (22,23). These authors also took into consideration nodules with a large amount of liquid, sometimes multi-RFA treatments were needed to achieve a significant volume reduction and the basal mean volume was significant lower compared to our study population one (22,23). In previous clinical trials (13,14,18,19,21), it has always been found that there is a significant amelioration of both symptoms and cosmetic score regardless of the thyroid nodule volume and location.

Conversely, we have shown that RFA is able to suddenly improve the symptom score in patients with medium or large nodules and this improvement remained stable until the end of the study period. However, no improvement in the symptom score was observed in the small nodule subgroup.

Moreover, we have shown that, in the overall study population, the cosmetic score improved significantly between baseline and 6 months and between 6 and 12 months while between 6 and 12 months it decreased significantly only in large group.

More than 20% of patients experienced local mild neck pain but it was not necessary to stop the treatment. Two people, a man and a woman, developed an autoimmune hyperthyroidism at 6 and 12 months after RFA treatment respectively. Graves’ disease or autoimmune hyperthyroidism is a complication that can be expected after laser ablation or PEI, probably due to the extensively damage of follicular thyroid cells. The mechanism for causality between these treatments and Graves’ disease is not completely known. Regalbuto and cols. had been issued a theory contend that the destruction of thyroid tissue after ablation therapy or injection of ethanol, among subjects genetically predisposed to autoimmune reactions, could release a large quantity of antigenic material (including TSHr protein) from follicular thyroid cells, that may trigger an autoimmune inflammatory response thought thyroid and orbital soft tissues (28). Anyway, we cannot surely state if, in these 2 subjects, the onset of autoimmune hyperthyroidism is due to the previous radiofrequency ablation treatment. One patient experienced major complication – permanent right paramedian vocal cord palsy with inspiratory stridor without dysphonia.

In our study, we did not perform a particular cost analysis comparing RFA to surgical procedure. Anyway, we agree with Bernardi’s findings (29). She has clearly shown that RFA may be a cost-effective technique to treat the thyroid nodules compared to surgery. Indeed, she performed a cost analysis included the procedures (RFA or surgery) and the respective pre- and post-procedural exams. The length of RFA session was short and was set in an outpatient regimen with an overall cost of about 1,660 € compared to surgical procedure (the operative time was longer, the length of the hospital stay was 1-2 days and the mean cost was about 4,550 €). However, a recent study (30) did not confirm these findings. Indeed, Che and cols. stated that compared with surgery, the advantages of radiofrequency ablation include fewer complications, preservation of thyroid function, and fewer hospitalization days but the cost difference was not significant.

The strength of our study is that compared to the previous published evidence, the study population is homogeneous regarding thyroid function, thyroid nodule volume, radiofrequency out-power and number of RFA sessions. In particular:

– We enrolled only patients with non-functioning thyroid nodules because it cannot be fully excluded that their functional state could affect the volumetric response to the RFA treatment.

– We previously excluded from our analysis patients with thyroid cystic nodule or thyroid nodules with a solid portion less than 70%.

– Thyroid nodules have been treated by one single RFA using the same radiofrequency outpower.

Furthermore, our mean baseline nodule volume is significant higher compared to the other studies (much more close to what we face in the the real life when we suggest the RFA technique). Moreover, compared to the previous publication (24), this study confirms the significant volume reduction at 6 months after RFA treatment and demonstrates further significant volume decline at 12 months after RFA in this homogeneous study population.

This study has a few major limitations. In particular, this is a retrospective study and the follow-up period is quite short.

In conclusion our study has shown that a single RFA treatment was effective in reducing benign thyroid nodules volume. Moreover, larger BTNs seem to be less responsive and perhaps in these cases a further RFA treatment should be used to get all the desired clinical and radiological outcomes. Cosmetic score improved in all treated BTNs, while symptom score got better only in medium and large BTN. RF ablation can be a valuable and generally safe tool for the non-surgical management of BTNs. Other large and prospective studies are needed to confirm these findings.
